# Bioinformatics Analysis Finds Immune Gene Markers Related to the Prognosis of Bladder Cancer

**DOI:** 10.3389/fgene.2020.00607

**Published:** 2020-06-23

**Authors:** Xingyu Chen, Yi Jin, Lian Gong, Dong He, YaXing Cheng, Mengqing Xiao, Yuxing Zhu, Zhanwang Wang, Ke Cao

**Affiliations:** ^1^Department of Oncology, Third Xiangya Hospital, Central South University, Changsha, China; ^2^Hunan Cancer Hospital, Xiangya School of Medicine, Central South University, Changsha, China

**Keywords:** bladder cancer, immune gene, prognosis, biomarker, tumor immune microenvironment

## Abstract

Bladder cancer is one of the most common malignant tumors of the urinary system that seriously threatens the health of a population. In recent years, the application of immunotherapy has significantly changed the treatment of bladder cancer, but only some patients can benefit from the treatment with immune-checkpoint inhibitors. Many problems are unsolved in the field of bladder cancer immunotherapy, especially in the search for genes that are critical to the level of immune cell infiltration and new effective therapeutic targets. We attempted to use bioinformatics analysis to identify immune gene markers related to the prognosis of bladder cancer and to establish a prognostic signature for bladder cancer patients based on their immune gene expression profiles. We used univariate Cox proportional hazards regression analysis, the least absolute shrinkage and selection operator (LASSO) Cox regression, and multivariate Cox proportional hazards regression analysis from The Cancer Genome Atlas bladder cancer cohort (TCGA-BLCA). Fifteen genes related to prognosis were screened using the survival analysis, correlation analysis, cancer and adjacent cancer differential expression analysis, and mutation analysis. The potential biological role of these genes was determined using survival analysis and principal component analysis (PCA). The receiver operating characteristic (ROC) curve assesses the prognostic value of the predictive signature. The gene ontology (GO), Kyoto Encyclopedia of Gene and Genome (KEGG), Gene set enrichment analysis (GSEA), and other methods were used to reveal the differential gene enrichment in the signaling pathways and cellular processes of high- and low-risk groups. The single-sample GSEA (ssGSEA) method was used to quantify the infiltration levels of 24 immune cells in the tumor immune microenvironment and these immune genes were found to be closely related to the tumor immune microenvironment. In summary, we screened 15 immune genes that were closely related to bladder cancer overall survival (OS) and may be potential prognostic indicators of bladder cancer. They may have research and clinical application value in bladder cancer immunotherapy. We used 15 immune genes to construct a new immune-related gene signature that was verified and could be helpful in improving individualized prognosis of patients with bladder cancer.

## Introduction

Bladder cancer is the ninth most common cancer worldwide. It has the characteristics of a difficult early diagnosis, rapid metastasis, and unsatisfactory treatment. In the past 10 years, the current treatment plan has not remarkably improved the 5-year survival rate, which needs to be addressed urgently. Additionally, finding effective biomarkers that assess and promote the diagnosis, treatment, and prognosis of bladder cancer (Dobruch et al., 2016) is important. To date, the prognosis of bladder cancer mainly depends on histopathological diagnosis and the tumor staging system. However, traditional methods are not sufficient to accurately evaluate the prognosis of bladder cancer patients and meet the needs of clinicians (Jordan and Meeks, 2019). Therefore, it is imperative to develop reliable and precise prognostic biomarkers to help clinicians optimize the treatment strategies.

The ICT is a treatment against CTLA-4, PD-1, or PD-L1. Recently, ICT has been applied to many aggressive cancers and it has changed the interventions for urinary cancers including advanced bladder cancer. The inhibition of the interactions between PD-1 and PD-L1 can restore the anti-tumor activity of the T cells and enhance the immune attack on the antigen (Ghasemzadeh et al., 2016; Kim, 2016). Bladder cancer is currently a highly immunogenic malignant tumor. In recent years, the ICT has achieved very good results in bladder cancer. In particular, the application of PD-1/PD-L1 inhibitors has greatly improved the incidence of benefit from survival for some patients. However, it is undeniable that only some of these patients can benefit from the treatment of immune checkpoint inhibitors as some patients do not respond to ICT or become resistant (Cheng et al., 2018; Felsenstein and Theodorescu, 2018; Schneider et al., 2019). Many problems remain to be solved in the bladder urothelial carcinoma (BUC) immunotherapy, especially in predicting immunotherapeutic biomarkers and finding new effective therapeutic targets.

Although several studies have proposed numerous biomarkers for predicting the efficacy of a treatment such as the expression of PD-L1, TMB, and microsatellite instability (MSI) biomarkers, most of these markers focus on the tumor invasion of the lymphocyte or TME. Disturbance in the immune response in a TME plays a decisive role in the development of bladder cancer. The constituent immune cells of a TME are an important part of the tumor tissue (Neal et al., 2018; Lemos et al., 2019). Many recent studies have shown that the effect of immune checkpoint inhibitors is affected by the tumor immune microenvironment that consists of effector CD8^+^, CD4^+^ cells, regulatory T cells, and dendritic cells (DCs) (Barry et al., 2018; Lambrechts et al., 2018; Sun et al., 2018). Improving the clinical response to an immune checkpoint blockade will require a deeper understanding of the factors affecting the local immune balance in the TME. Therefore, to explore the regulatory mechanism of the tumor immune microenvironment and the factors influencing the immune checkpoint inhibitors, we need to look for genes that are critical in affecting the level of the immune cell infiltration. Thus, targeted research and developmental interventions are of great significance for the diagnosis and treatment of bladder cancer.

In recent years, gene expression databases have been used to mine valuable therapeutic genes, identify promising prognostic factors, and analyze the molecular mechanisms of various cancers (Wei et al., 2019). Unlike the traditional individual molecular-prognostic-prediction indicators, a signature combining multiple genes can significantly improve the accuracy of prognosis prediction. Based on the importance of immune regulation in the diagnosis and treatment of bladder cancer and the general role of many immune genes in the prognosis of bladder cancer, we used single-factor Cox proportional hazards regression analysis to screen prognostic genes from the 314 immune-related genes in TCGA bladder cancer cohort (TCGA-BLCA). These genes were then subjected to the LASSO Cox regression and multi-factor Cox proportional hazard regression analyses to obtain 15 genes that would help establish the optimal risk signature. Survival analysis, correlation analysis, cancer and adjacent cancer differential expression analysis, and mutation analysis were carried out to explore the potential biological role of these genes. According to the median risk score, the patients were divided into the high-risk and low-risk groups. Survival analysis, PCA analysis, and ROC curve assessed the prognostic value of the risk scores. The GO and the KEGG databases were screened by the GSEA to explore the key signal pathway differences between the high-risk and low-risk populations. Finally, the ssGSEA method was used to quantify the infiltration levels of 24 immune cells in the tumor immune microenvironment and to explore the correlation between the 15 immune genes and the tumor immune microenvironment.

In conclusion, we screened 15 immune genes that were closely related to the overall survival (OS) of bladder cancer. They may prove to be potential prognostic indicators of bladder cancer, powerful predictors of immune checkpoint inhibitor responses, and/or new targets for immunotherapy. Simultaneously, we used 15 immune genes to construct and verify a new immune-related gene signature that could improve the individualized prognosis prediction of bladder cancer patients.

## Materials and Methods

### Database

The RNA-seq data and data on the clinical characteristics (including patient age, sex, stage, smoking status, and TNM staging) of the bladder cancer cohort were obtained from TCGA^1^ database.

### Selection of Immune-Associated Genes

The IMMUNE_RESPONSE and IMMUNE_SYSTEM_PROCESS 2 immune gene sets were obtained from the Molecular Signatures Database (MsigDB^2^) and 314 duplicate immune-related genes were removed. The mRNA expression data of these 314 genes were obtained from TCGA-BLCA.

### Identification and Validation of the Prognostic Gene Signature

The “survival” package of R language was used to perform the univariate Cox proportional hazards regression analysis to screen the immune genes that were significantly related to the OS of the TCGA-BLCA cohort. Using the “glmnet” package of R for the LASSO Cox regression analysis, a reduction in the dimensionality of high-dimensional data was achieved by limiting the sum of absolute values of the coefficients to less than a predetermined value. Variables with relatively small contributions were given coefficients of zero; only the genes with non-zero coefficients in the LASSO regression analysis were selected for further analysis. Finally, the obtained genes were subjected to multi-factor Cox proportional hazard regression analysis and screening to obtain 15 immune genes that would determine the best prediction signature are shown in Figure 3. These genes were selected to further calculate the risk score of each patient (Bao et al., 2014; Cheng et al., 2016).

riskScore=Expression×mRNA1Coefficient+mRNA1Expression×mRNA2Coefficient+mRNA2…ExpressionmRNAn×CoefficientmRNAn

According to the median value of the risk coefficient, the patients were divided into the high-risk and low-risk groups. The univariate Cox proportional hazard regression analysis and multivariate Cox proportional hazard regression analysis were performed on the risk value by using the “survival” package of the R language. Cox proportional hazard regression signature includes the risk score, age, gender, grade, T-, N-, and M-phase, and the smoking status. The Kaplan-Meier survival analysis was subsequently performed using the R “survival” package. The sensitivity and specificity of the ROC curve were used to evaluate the prognostic performance of the signature and the PCA was used to analyze the expression pattern of the grouped samples. A correlation analysis of the 15 immune genes was performed using the R “corrplot” package in the Pearson method and the results were displayed in the form of a Circos diagram are shown in Figure 4. The expression of these 15 immune genes was compared between cancer tissues and normal tissues. The mutations of the 15 immune genes in the TCGA-BLCA cohort were downloaded from the cBioPortal website^3^. The Kaplan-Meier survival analysis was performed on these 15 genes.

### Pathway Analysis

The “edgeR” package calculation in R language was used to perform a differential analysis of the mRNAs of the low-risk and the high-risk groups. To perform functional annotation from the GO^4^ for mRNAs with FDR values less than 0.05, the biological functions of differential genes, including biological processes (BPs), cellular components (CCs), and molecular functions (MFs) were analyzed. The KEGG^5^ database analyzes the metabolic pathways and signal transduction pathways in which differential genes are significantly enriched. A GSEA^6^ was then performed to reveal the signaling pathways and BPs in which differentially expressed genes were enriched between the high-risk and low-risk subgroups.

### Tumor Immune Microenvironment Analysis

#### Inference of Infiltrating Cells in the TME

Based on the immune cell marker genes provided by Bindea et al. (2013), a ssGSEA was used to quantify the infiltration levels of the 24 types of immune cells, including the T lymphocytes, DCs, and natural killer cells; the TCGA-BLCA (Finotello and Trajanoski, 2018) database was also used for this purpose. According to the level of immune cell infiltration, the patients were divided into the high-infiltration group and low-infiltration group. Heat maps were plotted to observe the relationship between risk value, age, T, N, and M stages, gender, and the immune infiltration levels of various immune cells in the high- and low-risk groups. Finally, the correlation between at-risk cells and immune cells was calculated using the Pearson method.

### Statistical Analysis

All analyses were performed using the R programming language^7^. Univariate and multivariate Cox proportional hazard regression analyses were also used to assess the relationship between the risk score and OS. The ROC analysis was used to detect the sensitivity and specificity of the genetic signature risk scores to predict survival. The area under the ROC curve (AUC) was used as an indicator of prognostic accuracy. In all analyses, *P*-values less than 0.05 were considered statistically significant.

## Results

### Construction of a Prognostic Signature for TCGA-BLCA

**FIGURE 1:**
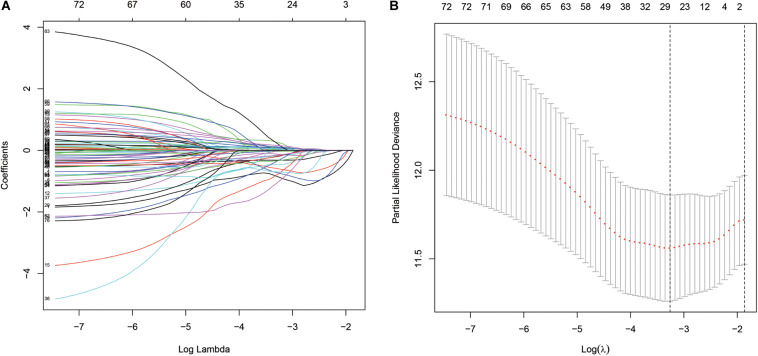
The least absolute shrinkage and selection operator (LASSO) Cox regression analysis. **(A)** LASSO coefficient profiles of the 76 immune-genes in TCGA-BLCA. **(B)** A coefficient profile plot was generated against the log (lambda) sequence.

**FIGURE 2:**
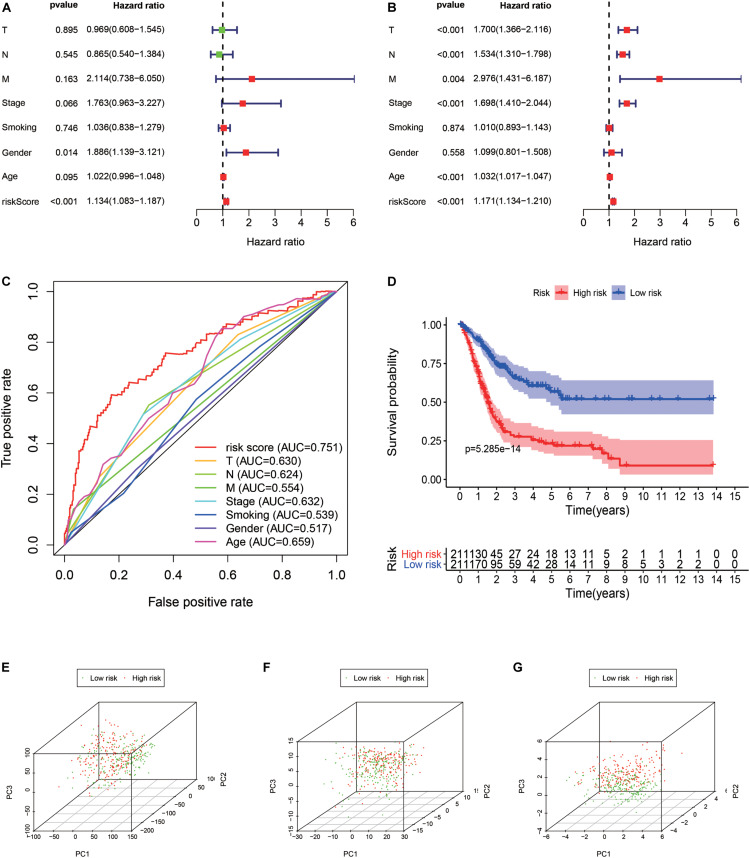
Signature validation. **(A)** Univariate Cox proportional hazards regression analysis and the **(B)** multivariate Cox proportional hazards regression analysis explored the correlation between the risk score, age, gender, grade, T, N, M-phases, smoking status, and the overall survival (OS). **(C)** The signature was evaluated by using the sensitivity and specificity of the ROC curve. **(D)** Kaplan-Meier analysis of TCGA bladder cancer patients stratified by median risk score. PCA analysis of the expression patterns of grouped samples using all genes **(E)**, 304 genes of the immune gene set **(F)**, and prognostic signature **(G)**.

A total of 314 immune-related genes were obtained from the MSigDB. Univariate Cox regression analysis was performed on these genes. Seventy-six immune genes from the TCGA-BLCA database were found to be significantly related to the OS. These genes were subjected to the LASSO regression analysis to calculate the correlation coefficients. The coefficients of each gene are shown in Figures 1A,B. Twenty-nine immune genes were obtained for the multi-factor Cox regression analysis. The signature performed best when only 15 genes were included. The results of the multi-factor Cox regression analysis for 15 genes are shown in Table 1. The expression and risk coefficient to calculate the risk value of each patient are indicated. The results of the univariate Cox proportional hazard regression analysis and multivariate Cox proportional hazard regression analysis showed that the riskScore was related to the OS of the TCGA-BLCA cohort (*P* < 0.01). The results of the multivariate Cox proportional hazard regression analysis showed that the T, N, and M stages, age, and riskScore were independent prognostic factors (Figures 2A,B). The ROC analysis detects the sensitivity and specificity of the genetic risk scores in predicting survival. The AUC of riskScore was 0.751 (Figure 2C), indicating that the signature displayed good sensitivity and specificity for predicting survival. The Kaplan-Meier survival analysis showed that the high-risk group had a lower prognosis than the low-risk group (*P* < 0.01, Figure 2D). The PCA results showed that our 15 immune genes could better divide the high- and low-risk patients into two groups compared with all other genes (Figure 2E) and the immune gene set 304 genes (Figure 2F). These results confirm the sensitivity and specificity of the signature.

**TABLE 1:** Multivariate COX regression analysis results of 15 immune genes.

	Multivariate Cox regression analysis					
Gene_symbol	Ensembl_ID	coef	HR	HR.95L	HR.95H	*p*-Value
CCR9	ENSG00000173585.15	−0.33390	0.71612	0.57411	0.89327	0.00307
HDAC7	ENSG00000061273.17	−0.39344	0.67473	0.50924	0.89401	0.00614
ZAP70	ENSG00000115085.13	−0.13452	0.87414	0.78843	0.96916	0.01062
IL7	ENSG00000104432.12	−0.18109	0.83436	0.72586	0.95908	0.01084
PTGER4	ENSG00000171522.5	−0.12127	0.88579	0.80315	0.97694	0.01524
CDK6	ENSG00000105810.9	0.12004	1.12754	1.01910	1.24752	0.01998
IL10	ENSG00000136634.5	0.13817	1.14817	1.01698	1.29628	0.02562
CTSG	ENSG00000100448.3	0.08613	1.08995	1.00825	1.17828	0.03027
CEBPG	ENSG00000153879.8	−0.32792	0.72042	0.53444	0.97113	0.03138
PF4	ENSG00000163737.3	0.15675	1.16971	1.00964	1.35515	0.03682
MAP3K7	ENSG00000135341.17	0.31106	1.36487	0.99807	1.86647	0.05143
ZBTB16	ENSG00000109906.13	0.07375	1.07653	0.99391	1.16603	0.07030
EREG	ENSG00000124882.3	0.04388	1.04486	0.99611	1.09599	0.07183
RUNX1	ENSG00000159216.18	−0.13852	0.87064	0.74289	1.02037	0.08710
CIITA	ENSG00000179583.17	−0.08344	0.91994	0.82220	1.02931	0.14542

**FIGURE 3:**
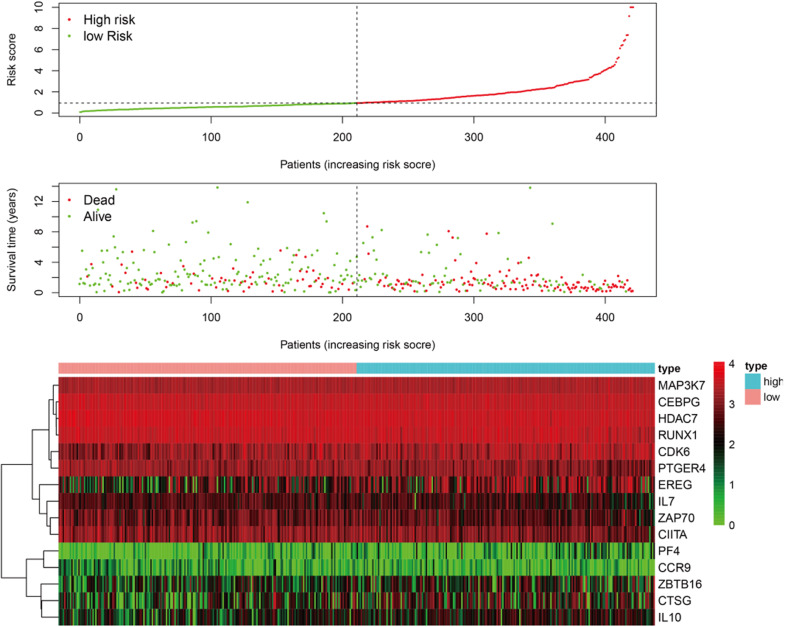
The patients were divided into two groups: low-risk and high-risk. As the risk score increased, the survival time of patients decreased and the number of deaths increased. The heat map shows the expression profile of the 15 immune genes in the prognostic markers.

**FIGURE 4:**
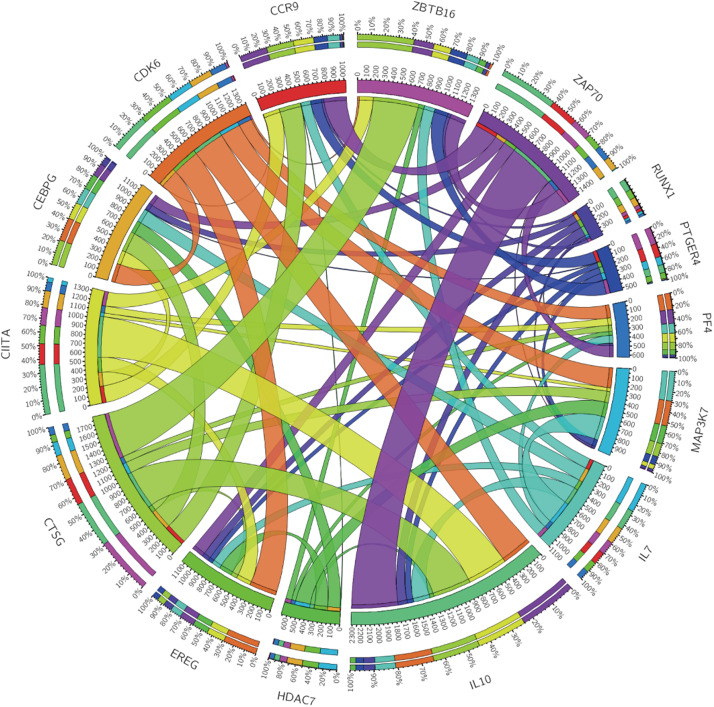
Circos plot showing the correlations between the 15 immune genes.

### Features of the Prognostic Signature

**FIGURE 5:**
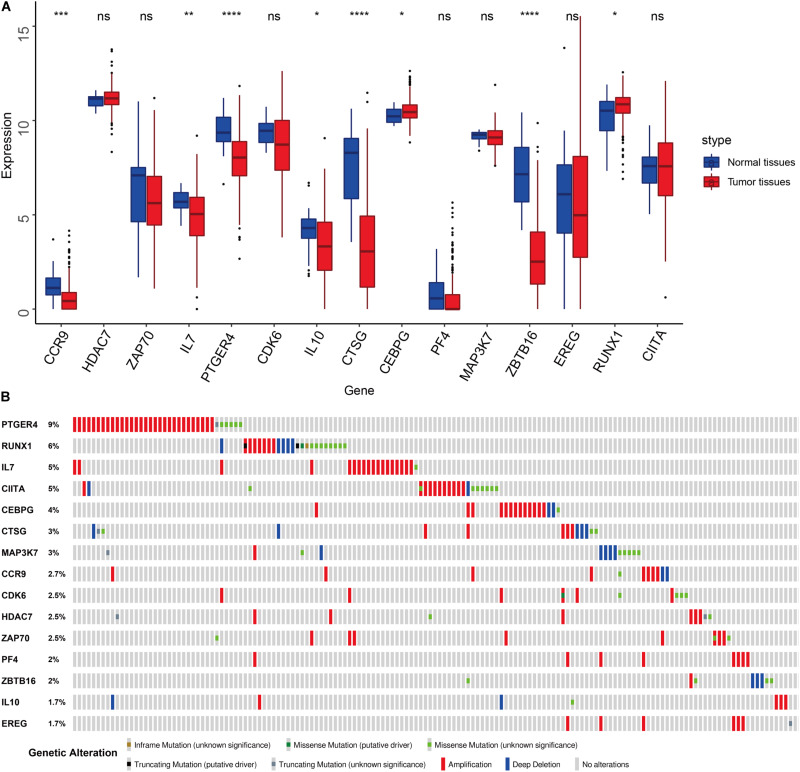
Features of the prognostic signature. **(A)** Differential expression of the 15 genes in the normal and cancer tissues of TCGA-BLCA cohort. **(B)** Mutations of 15 genes in the TCGA-BLCA cohort.

**FIGURE 6:**
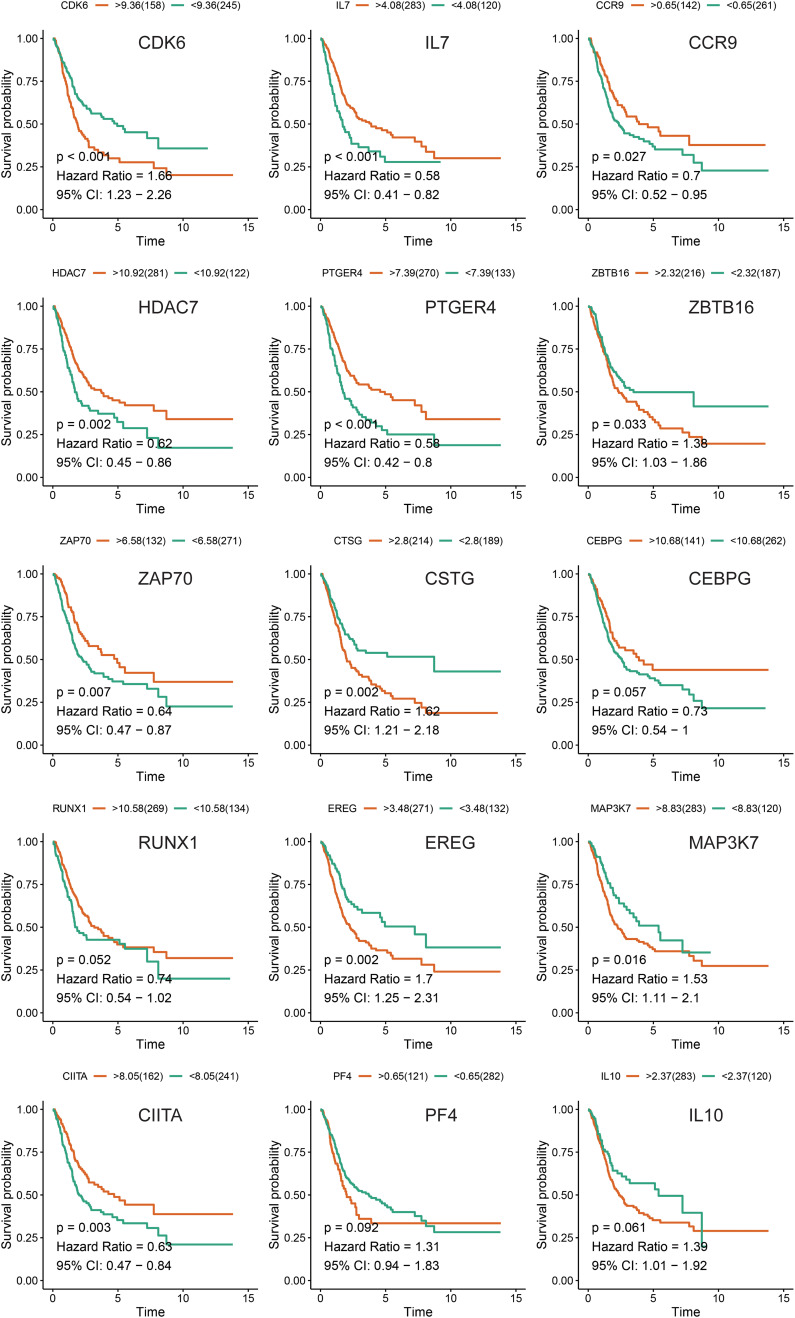
Kaplan-Meier survival analysis of the 15 immune genes in the TCGA-BLCA cohort.

We compared the expression of the 15 immune gene cancer tissues and adjacent tissues in the TCGA-BLCA cohort (Figure 5A) and found that the expression of the CCR9, IL7, PTGER4, IL10, CTSG, and ZBTB16 proteins in the adjacent tissues was significantly higher than that in the cancerous tissues (*P* < 0.05). The expression of CEBPG and RUNX1 proteins in the cancer tissues was significantly higher than that in the adjacent tissues (*P* < 0.05). Next, we examined the mutations in these genes in bladder cancer (Figure 5B) and found that the mutation frequency of PTGER4 was the highest, reaching 9%, with amplification mutations as the main mutation; it was followed by RUNX1, IL7, and CIITA, with mutation frequencies of 6, 5, and 5%, respectively. By analyzing the relationship between the 15 immune genes and the OS, it was found that the group showing a higher expression of the CDK6, HDAC7, CTSG, EREG, and ZBTB16 mRNAs had a lower survival time and was smaller. The group showing a higher expression of the ZAP70, IL7, and PTGER4 proteins had a significantly longer survival time than the lower expression group (*P* < 0.05, Figure 6).

### Identification of the Involved Signaling Pathways

**FIGURE 7:**
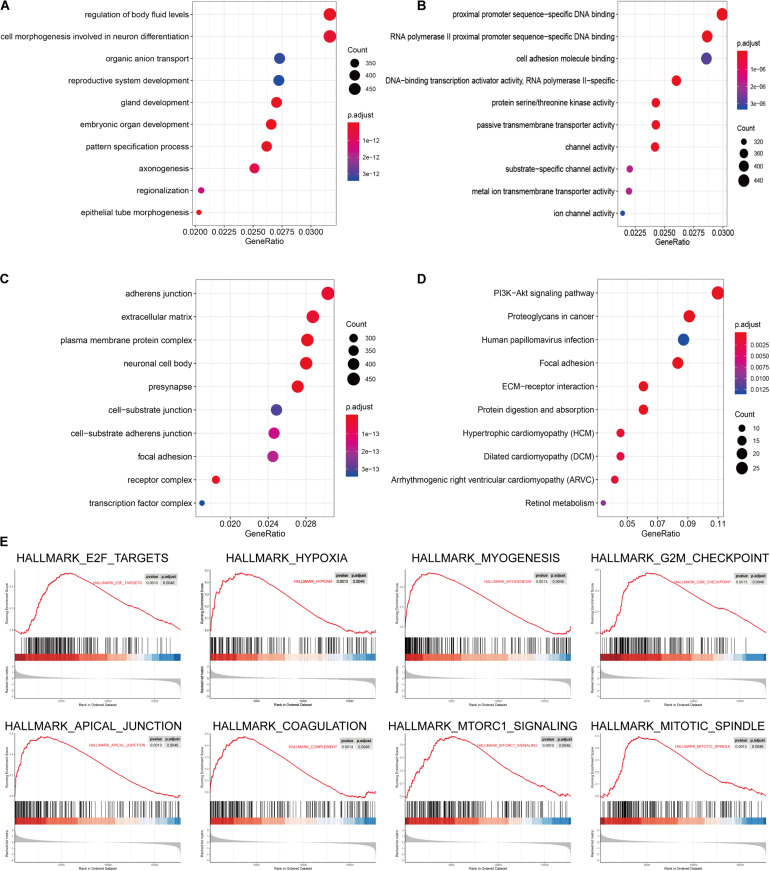
The signal pathway enrichment analysis was performed on the differential mRNA in the low-risk and high-risk groups. The GO analysis results consist of three parts: **(A)** biological process, **(B)** molecular function, and **(C)** cellular component. **(D)** Partial display of the KEGG analysis results. **(E)** Partial display of the GSEA analysis results.

The signaling pathway enrichment analysis of differential mRNA (FDR < 0.05) in the low-risk and high-risk groups and the GO analysis showed that the differential genes were related to the extracellular matrix organization, extracellular structure organization, and extracellular matrix. The collagen-containing extra cellular matrix, extra cellular matrix structural constituents, integral binding, and other BPs are closely related (Figures 7A–C). The KEGG analysis revealed that these differential genes were mainly enriched in the PI3K-Akt signaling pathway, in the proteoglycans in cancer, human papillomavirus infection, and other signaling pathways (Figure 7D and Table 2). The GSEA analysis showed that the signaling pathways such as E2F targets, hypoxia, G2/M DNA damage checkpoint, apical junction complex, epithelial–mesenchymal transition (EMT), KRAS Signaling UP, mTORC1 signaling, mitotic spindle, and TNFα signaling via NF-Kβ were significantly increased in the high-risk group (Figure 7E).

**TABLE 2:** KEGG analysis results of the differential genes in the low-risk and high-risk groups.

ID	Description	GeneRatio	BgRatio	*p*-Value	*p*.adjust	*q*-Value
hsa04512	ECM–receptor interaction	16/264	88/8011	2.12E-08	5.57E-06	5.08E-06
hsa05205	Proteoglycans in cancer	24/264	204/8011	5.03E-08	5.75E-06	5.25E-06
hsa04974	Protein digestion and absorption	16/264	95/8011	6.56E-08	5.75E-06	5.25E-06
hsa04510	Focal adhesion	22/264	199/8011	5.57E-07	3.66E-05	3.34E-05
hsa04151	PI3K-Akt signaling pathway	29/264	354/8011	4.81E-06	0.000253	0.000231
hsa05410	Hypertrophic cardiomyopathy (HCM)	12/264	90/8011	3.54E-05	0.001473	0.001344
hsa05412	Arrhythmogenic right ventricular cardiomyopathy (ARVC)	11/264	77/8011	3.92E-05	0.001473	0.001344
hsa05414	Dilated cardiomyopathy (DCM)	12/264	96/8011	6.78E-05	0.002229	0.002034
hsa00830	Retinol metabolism	9/264	67/8011	0.000321	0.009367	0.008548
hsa05165	Human papillomavirus infection	23/264	330/8011	0.000534	0.013218	0.012062
hsa00982	Drug metabolism − cytochrome P450	9/264	72/8011	0.000553	0.013218	0.012062
hsa00140	Steroid hormone biosynthesis	8/264	60/8011	0.000724	0.015869	0.014481
hsa00590	Arachidonic acid metabolism	8/264	63/8011	0.001007	0.020364	0.018583
hsa05144	Malaria	7/264	50/8011	0.001157	0.021738	0.019837
hsa00053	Ascorbate and aldarate metabolism	5/264	27/8011	0.001665	0.029199	0.026645
hsa05146	Amebiasis	10/264	102/8011	0.001881	0.030914	0.028211
hsa00860	Porphyrin and chlorophyll metabolism	6/264	42/8011	0.002341	0.036213	0.033046
hsa00980	Metabolism of xenobiotics by cytochrome P450	8/264	76/8011	0.003393	0.049577	0.045241

### The Prognostic Signature Is Related to the Tumor Immune Microenvironment

**FIGURE 8:**
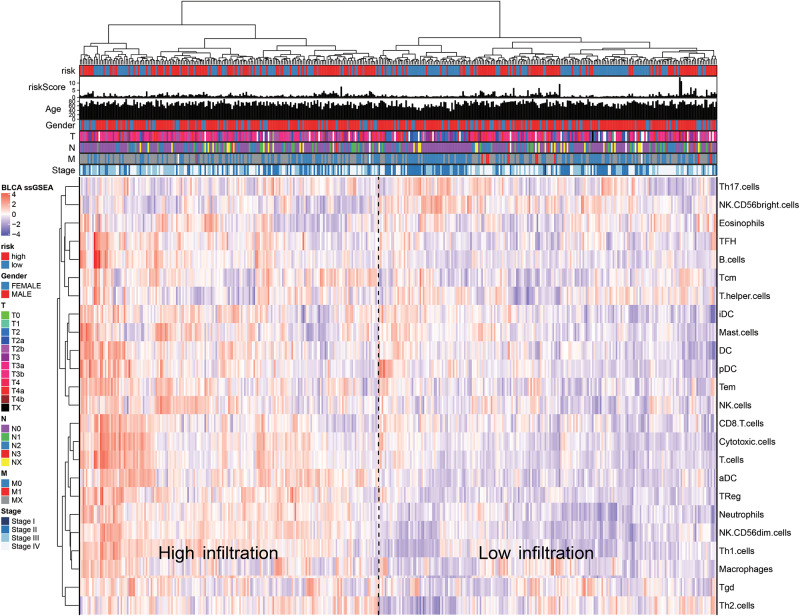
The ssGSEA method quantifies the level of invasion of the 24 immune cells in the tumor immune microenvironment. The composite heat map shows the relationship between the risk score, age, stage, T, N, and M stages, gender, and invasion of the 24 immune cells.

**FIGURE 9:**
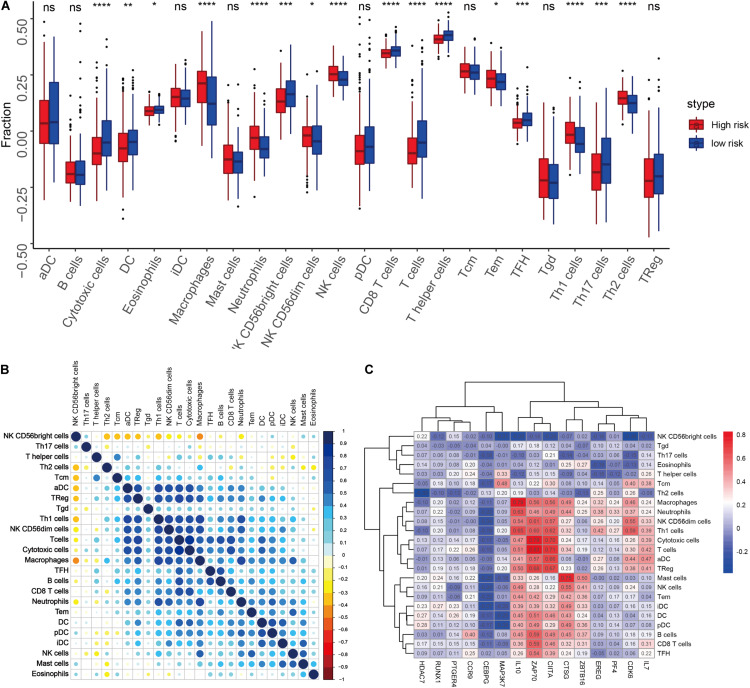
**(A)** Differences in the infiltration levels of the 24 immune cells in the high- and low-risk groups. **(B)** Correlations between the 24 immune cells. **(C)** Heat map of the correlation between the 15 immune genes and the 24 immune cells.

We quantified 24 types of immune cells including the B cells, T cells, natural killer cells, macrophages, DCs, and myeloid subpopulations to investigate the composition of the tumor immune microenvironment and draw a heat map to observe the risk values, age, stages T, N, and M, gender, and immune infiltration (Figure 8). At the same time, we found cytotoxic cells, DC, eosinophils, CD56^*bright*^ NK cells, CD8 T cells, T cells, and T helper cells in the high-risk group. The level of infiltration of TFH and Th17 cells was significantly lower than that in the low-risk group, while the levels of macrophages, neutrophils, CD56^dim^ NK cells, NK cells, Th1 cells, and Th2 cells in the high-risk group were significantly higher than that in the low-risk group (Figure 9A). At the same time, we analyzed the correlation between the immune cells. Among them, we focused on the significant positive correlation between CD8^+^ T cells closely related to the immune checkpoint inhibitors and iDC, DC, pDC, TReg, T cells, and cytotoxic cells (Figure 9B). Finally, we analyzed the correlation between the 15 immune genes and the immune cells and found that IL10, CIITA, ZAP70, and macrophages, neutrophils, CD56^dim^ NK cells, Th1 cells, cytotoxic cells, T cells, aDC, TReg, NK cells, Tem, iDC. The infiltration levels of the DC, pDC, B cells, CD8^+^ T cells, TFH, and other immune cells are positively correlated; the CEBPG has a negative correlation with the mast cells, NK cells, Tem T cells, Lentivirus-induced DCs (iDCs), and DC. The MAP3K7 has a positive correlation with the T helper cells and central memory T cells (Tcm cells) and a negative correlation with iDC, DC, and pDC. Cathepsin G is positively related to the mast cells, NK cells, Tem T cells, iDC, DC, plasmacytoid DCs (pDCs), B cells, macrophages, and neutrophils. CDK6 is positively correlated with Tcm cells, Th2 cells, macrophages, neutrophils, CD56^dim^ NK cells, Th1 cells, cytotoxic cells, T cells, aDC, and TReg and negatively correlated with NK CD56^*bright*^ cells. IL7 was positively correlated with the CD8^+^ T cells, Tcm cells, macrophages, neutrophils, CD56^dim^ NK cells, Th1 cells, cytotoxic cells, T cells, aDC, and TReg (Figure 9C).

## Discussion

Bladder cancer is the ninth most common cancer in the world, affecting 430,000 people and causing 165,000 deaths each year. Although considerable time, effort, and expense have been invested in bladder cancer research, the overall morbidity and mortality has not improved significantly over the past 20 years (Antoni et al., 2017). Modern treatments for bladder cancer include surgery, chemotherapy, radiation therapy, and immunotherapy. Immunotherapy brings a new hope in the treatment of bladder cancer. A large number of basic and clinical experiments are currently underway, including allogeneic stem cell transplantation, antitumor vaccines, proinflammatory cytokines, chimeric antigen receptors, and adoptive T cell metastasis. One of the most promising methods considered is ICT (Yu et al., 2018; Zhu et al., 2019; Zhuang et al., 2020).

In the human immune cycle of tumors, the antigens produced by the tumor cells are captured by the DCs. The major histocompatibility complex (MHC)-I and MHC-II on the surface of the DCs present these antigens to the T cells for recognition and lead to the activation of effector T cells (Ramachandran et al., 2017; Pio et al., 2019; Ryan et al., 2019). The internal and external environment of the tumor cells during tumorigenesis and metastasis is called the TME. The TME contains tumor cells and surrounding immune cells, endothelial cells, fibroblasts, extracellular matrix, secreted cytokines, chemokines, etc. Tumors can create a series of favorable conditions for themselves through the TME and even escape the immune cycle. In cancer patients, the tumor’s immune cycle does not perform well. The most important reason is that in the TME, there are some inhibitory signals that suppress the immune function of the effector T cells. The main role of the T cells is to distinguish healthy cells from pathogens or malignant cells by activating or deactivating various receptors on the surface of the T cells. The inhibitory signals include a class of signaling pathways called the immune checkpoints. These immune checkpoints are normally used to maintain the body’s autoimmune tolerance and prevent these killer T cells from attacking their own cells because these molecules and their relevant receptors on the T cells “control” the immune system by blocking the immune function, so they are collectively referred to as checkpoint proteins. In order to escape the hunting by T cells, some tumor cells also generate some inhibitory signals on their surface. The immune function of the T cells is suppressed by the immune checkpoints. As a result, the immune system remains inactive against malignant cells, allowing their uncontrolled growth and proliferation. The immune checkpoint inhibitors interfere with the action of these checkpoint proteins to prevent tumors from suppressing the T cells and restart the tumor immune cycle (Kieler et al., 2018; Li et al., 2019). Immune checkpoint inhibitors, in principle, should have a wide range of killing capabilities against cancer, but a large number of cases of non-response have been found in clinical applications (Ellmark et al., 2015; Coffelt and de Visser, 2016). Recent research indicates that non-response of immune checkpoint inhibitors may be related to various factors such as tumor immune microenvironment regulation, single immune checkpoint inhibitor suppression, and blocked T cell infiltration during the immune cycle. Therefore, the subsequent objectives include exploring the regulatory mechanism of the TME, clarifying the mechanism of immune checkpoint inhibitor non-response, and finding promising new targets for immunotherapy.

Gene markers, often used to predict prognosis, have been reported to be more accurate than the TNM staging methods in multiple cancer species (Bao et al., 2014; Liu et al., 2019). In this study, we screened genes related to prognosis from 314 immune-related genes. Using univariate Cox proportional hazards regression analysis, Lasso regression analysis, and multivariate Cox proportional hazards regression analysis, we finally obtained 15 independent prognostic immune genes. The survival analysis, correlation analysis, cancer and adjacent-cancer differential expression analysis, and mutation frequency analysis were performed on these genes. Some of these genes were closely related to the occurrence and development of bladder cancer: CDK6, IL-10, and RUNX1, of which CDK4/6 inhibitors are a promising treatment strategy for the treatment of bladder cancer (Zhao et al., 2015; Sun et al., 2019; Tong et al., 2019). The primary bladder tumor cells secrete a large amount of IL-10. Removing IL-10 in co-cultures of monocytes and tumor cells can reduce the upregulation of PD-L1 in monocytes and affect the curative effect of the immune checkpoint inhibitors (Wang et al., 2017). RUNX1 is a novel direct target of miR-27a, which is involved in the regulation of sensitivity to bladder cancer chemotherapy (Deng et al., 2015). There are few reports on the role of IL-7 and HDAC7 in bladder cancer. It was found that IL7 might be involved in T cell activation and play a role in the anti-CTLA-4 immunotherapy. Niegisch et al. (2013) showed that in urothelial carcinoma, the upregulation of the mRNAs of HDAC2 and HDAC8 and the downregulation of the mRNAs of HDAC4, HDAC5, and HDAC7 are common findings. The role of genes other than those mentioned here has not been reported in bladder cancer: CCR9, ZAP70, PTGER4, CTSG, CEBPG, PF4, MAP3K7, ZBTB16, CIITA, and EREG. These genes may be new markers for predicting the prognosis of bladder cancer and new targets for immunotherapy, but further basic and clinical laboratory identification is needed.

Signal pathway enrichment analysis of the differential mRNA (FDR < 0.05) in the low-risk and high-risk groups and the GO analysis results consisted of the CC, BP, and MF. At the CC level, the differential genes are related to the inheritance junction, extracellular matrix, and plasma membrane protein complex. At the MF level, the differential genes are related to promoter promoter-specific DNA binding, RNA polymerase II proximal promoter sequence-specific DNA binding, etc. At the BP level, the differential genes are related to the regulation of body fluid levels, cell morphogenesis involved in neuron differentiation, etc. These pathways are closely related to the BPs of tumorigenesis and development. KEGG analysis found that these differential genes were mainly enriched in the classical cancer signaling pathways such as the PI3K-Akt signaling pathway, proteoglycans in cancer, human papillomavirus infection, and focal adhesion (Figure 7D and Table 2).

For example, the PI3K-Akt signaling pathway has been studied to confirm that when its function is normal, the PI3K pathway regulates key cellular functions, including cell growth, movement, proliferation, and differentiation. However, excessive activation of the PI3K signaling pathway causes breast cancer and ovarian cancer (Shukla et al., 2007). New research shows that the PI3K-Akt signaling pathway plays an important role in the tumor immune microenvironment, affecting the efficacy of the immune checkpoint inhibitors (Li et al., 2018; Cristofoletti et al., 2019). The GSEA analysis found that the proteins in the high-risk group are positively correlated with hypoxia, EMT, myogenesis, E2F targets, G2/M checkpoint, apical junction, KRAS signaling up, mTORC1 signaling, mitotic spindle, complement, inflammatory response, TNFα signaling via NFKβ, apoptosis, coagulation, UV response signal pathways such as Dn and angiogenesis, and negatively correlated with the interferon α response signal pathway (Figure 7E and Table 3). Most of these pathways are closely related to tumorigenesis and the regulation of the TME. For example, hypoxia is a common feature of malignant tumors. It can regulate the tumor immune microenvironment by regulating a variety of immune cells. Hypoxia significantly reduces the T lymphocyte proliferation and activation, decreases the NKG2D receptor on the NK cells, and thereby inhibits the killing function of the NK cells, increases tumor-associated macrophages to induce angiogenesis, and reduces inflammation to promote tumor progression (Marques et al., 2012; Tian et al., 2017; Jiao et al., 2019). The EMT signaling pathway is an important BP for the epithelial-derived malignant tumor cells to acquire the ability to migrate and invade. It is of great significance in the occurrence, development, and metastasis of bladder cancer and participates in the TME regulation (Zhaojie et al., 2019). Interferons play a vital role in the immune response of the body toward malignant cells. Type I interferons (IFNα and IFNβ) directly regulate the transcription of more than 100 downstream genes, resulting in countless direct (via cancer cells) and indirect (via immune effector cells and vasculature) on tumors. The IFN-α/β receptor (IFNAR) signaling can promote the Treg function in autoimmunity. Activation of the IFNα signaling pathway leads to a more effective antiviral response and enhanced antitumor immunity (Gangaplara et al., 2018; Borden, 2019). In our study, the high-risk group was negatively correlated with the interferon alpha response signal pathway (NES = −1.427, *P*.adjust = 0.041, Table 3). Finally, we quantified the levels of 24 immune cell infiltrations in TCGA-BLCA tumor samples using the ssGSEA method. On comparing these levels in the high- and low-risk groups, we found that the levels of the cytotoxic cells, DC, CD8^+^ T cells, T cells, the T helper cells, TFH, Th17 cells, CD56^*bright*^ NK cells, and eosinophils were significantly lower than that in the low-risk group. It is well known that the higher the infiltration levels of the cytotoxic cells, DCs, CD8^+^ T cells, T cells, and T helper cells in a patient’s tumors, the greater the survival benefit for the patients. The cell infiltration level groups such as DC, CD8^+^ T cells, T cells, and T helper cells affect the efficacy of ICT in a positive manner (Jiao et al., 2019). This may partly explain the phenomenon that the survival time of the high-risk group is significantly lower than that of the low-risk group (Figure 9A). The above results further suggest the reliability of the prediction signature, its relevance to the TME, immunological examination, and the importance of the 15 immune genes. We then analyzed the correlation between these 15 immune genes and immune cells. We observed that most of the immune genes have a high correlation with the level of immune cell infiltration in the TME, but the specific mechanism needs further experimental investigation.

**TABLE 3:** GSEA analysis of the differential genes in the low-risk and high-risk groups.

Description	setSize	enrichmentScore	NES	*p*-Value	*p*.adjust	*q*-Values
HALLMARK_E2F_TARGETS	189	0.52760	1.93416	0.00127	0.00455	0.00220
HALLMARK_HYPOXIA	190	0.48029	1.75966	0.00127	0.00455	0.00220
HALLMARK_MYOGENESIS	199	0.54299	1.98919	0.00127	0.00455	0.00220
HALLMARK_G2M_CHECKPOINT	188	0.57140	2.08917	0.00128	0.00455	0.00220
HALLMARK_APICAL_JUNCTION	194	0.57149	2.08904	0.00129	0.00455	0.00220
HALLMARK_EPITHELIAL_MESENCHYMAL_TRANSITION	194	0.81607	2.98307	0.00129	0.00455	0.00220
HALLMARK_KRAS_SIGNALING_UP	194	0.51106	1.86815	0.00129	0.00455	0.00220
HALLMARK_MTORC1_SIGNALING	194	0.46253	1.69076	0.00129	0.00455	0.00220
HALLMARK_MITOTIC_SPINDLE	196	0.49540	1.81105	0.00129	0.00455	0.00220
HALLMARK_COMPLEMENT	195	0.47718	1.74348	0.00129	0.00455	0.00220
HALLMARK_INFLAMMATORY_RESPONSE	197	0.54348	1.98680	0.00129	0.00455	0.00220
HALLMARK_TNFA_SIGNALING_VIA_NFKB	197	0.54198	1.98130	0.00129	0.00455	0.00220
HALLMARK_APOPTOSIS	159	0.46992	1.68354	0.00132	0.00455	0.00220
HALLMARK_COAGULATION	136	0.55307	1.93471	0.00136	0.00455	0.00220
HALLMARK_UV_RESPONSE_DN	137	0.58042	2.03005	0.00137	0.00455	0.00220
HALLMARK_ANGIOGENESIS	36	0.72246	2.04503	0.00152	0.00473	0.00229
HALLMARK_MYC_TARGETS_V1	192	0.41148	1.50503	0.00256	0.00716	0.00347
HALLMARK_IL2_STAT5_SIGNALING	195	0.42002	1.53464	0.00258	0.00716	0.00347
HALLMARK_IL6_JAK_STAT3_SIGNALING	87	0.47494	1.56316	0.00289	0.00762	0.00369
HALLMARK_HEDGEHOG_SIGNALING	35	0.58977	1.66473	0.00456	0.01140	0.00552
HALLMARK_UV_RESPONSE_UP	154	0.40707	1.45416	0.00653	0.01554	0.00753
HALLMARK_UNFOLDED_PROTEIN_RESPONSE	109	0.42188	1.44553	0.01094	0.02487	0.01204
HALLMARK_INTERFERON_ALPHA_RESPONSE	93	−0.38276	−1.42683	0.01899	0.04128	0.01999

## Conclusion

In summary, we screened 15 immune-related markers that have independent prognostic significance for bladder cancer. They may be used as potential prognostic indicators of bladder cancer and related to the level of tumor cell microenvironment immune cell infiltration. We hope to provide an additional feasible method for assessing the prognosis of bladder cancer and may provide valuable new targets for anti-tumor immunotherapy.

## Data Availability Statement

Publicly available datasets were analyzed in this study. These can be found in The Cancer Genome Atlas (https://portal.gdc.cancer.gov/).

## Author Contributions

All authors listed have made a substantial, direct and intellectual contribution to the work, and approved it for publication.

## Conflict of Interest

The authors declare that the research was conducted in the absence of any commercial or financial relationships that could be construed as a potential conflict of interest.

## Footnotes

1 https://portal.gdc.cancer.gov/

2 https://www.gsea-msigdb.org/gsea/msigdb/search.jsp

3 https://www.cbioportal.org/

4 http://geneontology.org/

5 https://www.genome.jp/kegg/

6 http://software.Broadstitute.org/GSEA/

7 https://www.r-project.org/

## Abbreviations

CTLA-4: cytotoxic lymphocyte antigen-4
GO: gene ontology
GSEA: gene set enrichment analysis
ICT: immune checkpoint therapy
KEGG: Kyoto Encyclopedia of Gene and Genome
LASSO: least absolute shrinkage and selection operator
PCA: principal component analysis
PD-1: programmed cell death protein 1
PD-L1: programmed-death ligand 1
ROC: receiver operating characteristic
SsGSEA: single-sample gene set enrichment analysis
TCGA: The Cancer Genome Atlas
TMB: tumor mutation burden
TME: tumor microenvironment.
